# Neoadjuvant concurrent chemoradiation with weekly paclitaxel and carboplatin for patients with oesophageal cancer: a phase II study

**DOI:** 10.1038/sj.bjc.6603134

**Published:** 2006-05-02

**Authors:** E van Meerten, K Muller, H W Tilanus, P D Siersema, W M H Eijkenboom, H van Dekken, T C K Tran, A van der Gaast

**Affiliations:** 1 Department of Medical Oncology, Erasmus MC - University Medical Centre Rotterdam, The Netherlands; 2 Department of Radiotherapy, Erasmus MC - University Medical Centre Rotterdam, The Netherlands; 3 Department of Surgery, Erasmus MC - University Medical Centre Rotterdam, The Netherlands; 4 Department of Gastroenterology and Hepatology, Erasmus MC - University Medical Centre Rotterdam, The Netherlands; 5 Department of Pathology, Erasmus MC - University Medical Centre Rotterdam, The Netherlands

**Keywords:** oesophageal carcinoma, chemotherapy, chemoradiotherapy, combined modality treatment

## Abstract

This study was performed to assess the efficacy and safety of preoperative chemoradiation consisting of carboplatin and paclitaxel and concurrent radiotherapy for patients with resectable (T2-3N0-1M0) oesophageal cancer. Treatment consisted of paclitaxel 50 mg m^−2^ and carboplatin AUC=2 on days 1, 8, 15, 22 and 29 and concurrent radiotherapy (41.4 Gy in 23 fractions, 5 days per week), followed by oesophagectomy. All 54 entered patients completed the chemoradiation without delay or dose-reduction. Grade 3–4 toxicities were: neutropaenia 15%, thrombocytopaenia 2%, and oesophagitis 7.5%. After completion of the chemoradiotherapy 63% had a major endoscopical response. Fifty-two patients (96%) underwent a resection. The postoperative mortality rate was 7.7%. All patients had an R0-resection. The pathological complete response rate was 25%, and an additional 36.5% had less than 10% vital residual tumour cells. At a median follow-up of 23.2 months, the median survival time has not yet been reached. The probability of disease-free survival after 30 months was 60%. In conclusion, weekly neoadjuvant paclitaxel and carboplatin with concurrent radiotherapy is a very tolerable regimen and can be given on an outpatient basis. It achieves considerable down staging and a subsequent 100% radical resection rate in this series. A phase III trial with this regimen is now ongoing.

The prognosis of oesophageal cancer is poor in symptomatic patients, for example, those with dysphagia. At the time of first diagnosis, almost half of such patients already have metastatic disease; the other half usually has locally advanced disease (T3N0 or T3N1). Furthermore, although surgical resection is still the first choice of treatment for fit patients with resectable disease, most of these patients have a poor outcome. This is reflected by a 5-year survival rate of approximately 20% (Hulscher *et al*, 2001). Despite the routine use of staging procedures such as computed tomography (CT), magnetic resolution imaging (MRI) and endoscopic ultrasound (EUS), many oesophageal tumours are incompletely resected (van Meerten and van der Gaast, 2005). In a number of large randomised studies, the percentage of incomplete resections varied between 25 and 46% (Kelsen *et al*, 1998; Hulscher *et al*, 2002; MRC, 2002). Hulscher *et al* found an incomplete resection rate of 25% in extended transthoracic surgery *vs* 29% in limited transhiatal resection for adenocarcinoma of the oesophagus. In the study performed by the Medical Research Counsel, resection was microscopically complete (R0) in 60% when surgery was preceded by chemotherapy, and 54% in the surgery alone group. Kelsen *et al* found incomplete resections in 14% of the patients when surgery was preceded by chemotherapy *vs* 30% for surgery alone.

Preoperative chemoradiotherapy may induce considerable tumour shrinkage and thereby increase the number of radical resections. In this setting, concurrent chemoradiotherapy with 5-fluorouracil (5-FU) and cisplatin is one of the most commonly used regimens. Unfortunately, the impact of preoperative chemoradiotherapy with 5-FU and cisplatin on survival is uncertain. An improved 3-year survival was shown in three meta-analyses of randomised controlled trials comparing neoadjuvant chemoradiotherapy and surgery to surgery alone (Kaklamanos *et al*, 2003; Urschel and Vasan, 2003; Fiorica *et al*, 2004), but if the study by Walsh *et al* (1996) is excluded, this benefit is lost. Furthermore, chemoradiotherapy with 5-FU and cisplatin can induce severe toxicity and most patients have to be hospitalised for this treatment. Thus, the best regimen of preoperative chemoradiation has not yet been established.

Recently, studies with radiotherapy combined with paclitaxel with or without cisplatin or carboplatin have shown promising results in other tumour types. Paclitaxel is a microtubule-stabilising agent that blocks the cell cycle in the G2 and M phase, the most radiosensitive phase. The radioenhancing effects of paclitaxel have been demonstrated *in vitro* in a human leukaemic cell line and in cell lines of squamous cell carcinoma and astrocytoma (Tishler *et al*, 1992; Choy *et al*, 1993; Leonard *et al*, 1996). Besides its radiosensitising effect, it also enhances the result of radiotherapy by increasing apoptosis and tumour reoxygenation. A weekly schedule permits an increase in dose-intensity and can provide continuous radiosensitizing plasma drug levels.

The combination of paclitaxel and carboplatin with concurrent radiotherapy has been tested in patients with advanced non-small-cell lung cancer. In five phase II studies, the combination of paclitaxel and carboplatin was given weekly with concurrent radiotherapy, followed by two or four 21-day cycles of consolidation chemotherapy (Choy *et al*, 1998, 2000; Lau *et al*, 2001; Ratanatharathorn *et al*, 2001; Kaplan *et al*, 2004). The overall response rate varied from 71 to 79%. The major toxicity was oesophagitis. In 10–46% of the patients a grade 3 or 4 oesophagitis was found. Treatment with paclitaxel and carboplatin and concurrent radiotherapy can be given on an outpatient basis, which is advantageous. Furthermore, this regimen is probably less toxic than cisplatin-based therapy.

On the basis of these considerations, we initiated a phase II study to determine the response rate and toxicity of a preoperative chemoradiotherapy regimen consisting of carboplatin and paclitaxel with concurrent radiotherapy in patients with a potentially resectable carcinoma of the oesophagus.

## METHODS

### Eligibility criteria

Patients with histologically proven squamous cell carcinoma, adenocarcinoma, or undifferentiated carcinoma of the oesophagus with the upper border at least 3 cm below the upper oesophageal sphincter were included. Disease was limited to T1N1 or T2-3N0-1M0 tumours. Tumours extending below the gastro-oesophageal (GE) junction into the proximal stomach were also eligible, provided that the bulk of the tumour was located in the oesophagus. The longitudinal tumour length had to be ⩽8 cm and the radial tumour length ⩽5 cm. Patients were required to be aged 18–75 years and to have an Eastern Cooperative Oncology Group (ECOG) performance status of ⩽2. Other criteria included adequate haematological, renal, hepatic and pulmonary functions as defined by: a granulocyte count of at least 1500 mm^−3^ and a platelet count 100 000 mm^−3^; a serum creatinine level <120 *μ*mol l^−1^ and a bilirubin level ⩽1.5 × upper normal limit; and a forced expiratory volume in one second (FEV1) of at least 1.2 l. The Medical Ethics Committee of Erasmus University Medical Centre approved the study. Written informed consent was required. No previous chemotherapy and radiotherapy or a past or current history of malignancy other than entry diagnosis was allowed, except for non-melanomatous skin cancer, curatively treated carcinoma *in situ* of the cervix, or a ‘cured’ malignancy more than 5 years before enrolment. Patients were not eligible if they had lost more than 10% of their body weight or had an inadequate caloric- and/or fluid intake.

### Staging

Pretreatment evaluation included a detailed history taking, a physical examination and a routine complete blood work-up. All patients underwent a baseline upper gastrointestinal (GI) endoscopy and EUS, and a CT of the chest and the upper abdomen, plus ultrasonography of the neck and pulmonary function tests.

### Treatment

#### Chemotherapy

Paclitaxel 50 mg m^−2^ and Carboplatin targeted at an AUC of 2 were administered on days 1, 8, 15, 22 and 29. All patients received dexamethasone 10 mg, clemastine 2 mg and ranitidine 50 mg, administered intravenously 30 min before paclitaxel infusion. Paclitaxel was given as a 1-h infusion diluted in 500 ml of sterile and isotonic sodium chloride solution (saline). After the completion of the paclitaxel infusion, 100 ml of saline was infused over 30 min followed by an infusion of 8 mg ondansetron or its equivalent diluted in 100 ml of saline given over 30 min. Hereafter, the total calculated dose of carboplatin diluted in 500 ml of 5% dextrose solution was administered over 1-h. Dose modifications were made for toxicity, using the National Cancer Institute – Common Toxicity Criteria (NCI-CTC version 2).

#### Radiotherapy

All patients were irradiated by external beam radiation, using a 3-D conformal radiation technique. The gross tumour volume (GTV) was defined by the primary tumour and any enlarged regional lymph nodes, and was drawn on each relevant CT slice. The planning target volume (PTV) provided a 1.5 cm radial margin and a proximal and distal margin of 4 cm around the GTV. If the tumour extended into the stomach, a distal margin of 3 cm was chosen. Before the start of the irradiation, a planning CT scan was made from the cricoid to L1 vertebra with a slice thickness of ⩽5 mm, with the patient in treatment position. Beams-eye-view (BEV) was used to ensure optimal target volume coverage and optimal normal tissue sparing. The prescription dose was specified at the ICRU 50/62 reference point, which was the isocenter for most patients. The daily prescription dose was 1.8 Gy at the ICRU reference point and the 95% isodose had to encompass the entire planning target volume (PTV). The maximum to the PTV was not allowed to exceed the prescription dose by >7% (ICRU 50/62) guidelines. Tissue density inhomogeneity correction was used. Portal images were obtained during the first fraction of all fields. A total dose of 41.4 Gy was given in 23 fractions of 1.8 Gy, with 5 fractions per week starting on the first day of the first cycle of chemotherapy.

#### Surgery

Surgery was planned within 6 weeks after the completion of the chemoradiation. For carcinomas located distally of the tracheal bifurcation, a transhiatal oesophageal resection was favoured. For carcinomas located proximally to the tracheal bifurcation, a transthoracic oesophageal resection was performed. In both techniques, a wide local excision including the N1 lymph nodes was carried out, including a standard resection of the lymph nodes around left gastric artery. The continuity of the digestive tract was restored by means of a gastric tube reconstruction with an anastomosis in the neck.

### Pathological analysis

The resection specimen was evaluated using a standard protocol, providing information on margins, tumour type, extension of the tumour, and lymph nodes. The sixth edition of the International Union Against Cancer (UICC) was used for TNM-classification, tumour grade, and stage grouping (UICC, 2002). When no tumour tissue could be seen, lesions such as an ulcer or an irregular area covered by mucosa were embedded in total together with surrounding areas in order to adequately judge the presence of residual tumour and therapy effects. The grading of the therapy response was performed as described by Junker *et al* (1997). The degree of histomorphological regression, that is, the effect of chemoradiation, was classified into four categories: grade I: more than 50% vital residual tumour cells; grade II: 10–50% vital residual tumour; grade III: less than 10% vital residual tumour cells; grade IV: complete tumour regression, no evidence of vital tumour cells.

A two-field lymph node dissection was carried out containing regional (mediastinal, oesophageal) and distant sites (coeliac region). The resection margins, especially the circumferential margin, were evaluated with a 1 mm cutoff point for vital tumour, implying that the tumour-free margin is >1 mm. If vital tumour was present at ⩽1 mm from the surgical resection margin it was considered positive.

### Restaging and follow-up

Upper GI endoscopy and CT of the chest and upper abdomen were repeated after the completion of the chemoradiation and ahead of the planned operation. Pulmonary function tests were repeated 6 and 12 months after therapy. Follow-up visits were performed every 3 months during the first 2 years and every 6 months thereafter to document late toxic effects, and, if applicable, disease relapse or progression, and death.

### Statistical analysis

As the statistical design was intended to allow us to detect a response percentage of at least 40%, it was calculated that 50 patients were needed. The pathological response to chemoradiation was defined as mentioned above. The response to chemoradiotherapy evaluated by endoscopy was classified as either no response, less than 50% response, more than 50% response, or complete response. These broad classifications were used in an attempt to reduce inter-observer variation (Brown *et al*, 2004). Tumour response evaluated by radiology was assessed according to the response evaluation criteria in solid tumours (RECIST) (Therasse *et al*, 2000).

Survival time was calculated as the duration from the day of start of chemoradiotherapy to death or the last follow-up, and recurrence-free interval was calculated from the day of surgery to the day of diagnosis of recurrence. Overall and disease-free survivals were estimated using the Kaplan–Meier method. Median survival time was obtained from the time corresponding to 50% survival based on the Kaplan–Meier survival curve.

## RESULTS

### Patient characteristics

Fifty-four eligible patients were enrolled between February 2001 and January 2004. Written informed consent was obtained from all patients. Characteristics of these 54 patients are summarised in Table 1. Of all patients, 91% were male and 76% had an adenocarcinoma. Of these 41 adenocarcinomas, 23 were located at the gastro-oesophageal junction and 18 in the distal oesophagus. The patient with 12% weight loss was accepted for chemoradiotherapy, because the weight loss was partly due to *de novo* diagnosed diabetes mellitus.

### Toxicity of and adherence to chemoradiotherapy

Fifty-three patients (98%) completed the preoperative treatment. One patient died at home after the second course of chemotherapy, probably due to a cardiac arrest. All other patients completed the neoadjuvant chemoradiotherapy as scheduled, without treatment delay or dose reduction. The 1.5 cm radial radiation margin was achieved in all patients; there were no compromises. The V20 was obtained in all patients and never exceeded 30% of the total lung volume. The acute toxicities due to the chemoradiation were usually mild. Haematological toxicity is listed in Table 2. Grade 3 or 4 toxicity consisted of leucopaenia in 13 patients (23.5%), neutropaenia in 8 patients (15.1%), and thrombocytopenia in 1 patient (1.9%). Two patients required a blood transfusion for anaemia (3.8%). Infectious complications were rare, only one patient was treated for pneumonia due to aspiration, no neutropenia was found. Three other patients also developed fever, but no infectious focus was found (see below). Relevant non-haematological toxicity (Table 3) consisted mainly of oesophagitis and dysphagia. Four patients (7.5%) developed grade 3 oesophagitis. Dysphagia improved during chemoradiation in 17 of 35 patients (48.6%), three of whom had initial nutritional support, because of grade 3 dysphagia, which could be discontinued. Dysphagia worsened in nine patients (17%), three of whom required nutritional support because of grade 3 dysphagia. In three patients needing nutritional support before starting chemoradiation the support could not be discontinued during the chemoradiation. Sensory neuropathy was seen in only five patients (9.4%), and in three patients it resolved after completion of the chemoradiotherapy. Seven patients (13.2%) were hospitalised. Five of them were briefly hospitalized for placing a nasogastric tube for nutritional support, because of grade 3 oesophagitis or dysphagia. One also had a pulmonary embolism and fever with grade 3 neutropaenia, one was also treated for pneumonia due to aspiration, and one patient also had fever with grade 3 neutropenia. One patient was hospitalised because of rectal bleeding. Colonoscopy revealed a non-malignant polyp, which was removed. One patient was hospitalised because of vomiting and fever with grade 2 neutropaenia.

### Response to chemoradiotherapy

Evaluation with upper GI endoscopy and CT was done after a mean of 10 days following the last radiotherapy session. Response evaluation with endoscopy showed a complete response in 10 patients (18.9%), a major response in 23 patients (43.4%), a minor response in 11 patients (20.8%), and no response in 1 patient (1.9%). In five patients response evaluation by endoscopy was not possible (in one patient there was ‘no pass’, in four patients the baseline endoscopy was performed in another hospital) and in three patients no endoscopy was performed after completing the chemoradiation. Response evaluated with CT showed no complete response or disease progression. In three patients (5.6%) a partial response was observed.

### Surgical results

One patient refused surgery after having completed the chemoradiotherapy. Endoscopy in this patient revealed a complete response. After 12 months of follow-up, a local recurrence of the oesophageal tumour was diagnosed, further workup also revealed supraclavicular lymph nodes. He refused further treatment. Thirteen months later he died from progressive disease. A transhiatal oesophagectomy was performed in 46 of the 52 patients, and a transthoracic resection in 6 patients. The median time between the completion of chemoradiotherapy and surgery was 42 days (range 20–74 days). The in-hospital postoperative mortality rate was 7.7% (CI 0–15%). Two patients died from systemic complications due to anastomotic leakage, one patient from a cerebral vascular accident one day after surgery, and one from sepsis. Autopsy in the latter patient revealed a prostatitis as the probable focus of the sepsis. Postoperative complications were seen in 38 patients (73%). These complications were mainly pulmonary (42%) or cardiac (13%) (Table 4). Besides the two lethal anastomotic leaks, in 10 of the 48 (20.8%) patients surviving postoperatively an anastomotic leak was seen. In five of them (10.4%) the leakage was a radiological finding on routinely performed contrast swallow postoperatively. In all patients the clinical signs of the leak could be treated conservatively, but in two patients (4.2%) the leak resulted in long-term nutritional support. Twenty-two patients (45.8%) developed an anastomotic stricture requiring endoscopic dilatation (range 1–27, median 7). Eventually, all patients were able to eat solid food.

### Pathological results

In 13 patients no residual tumour in the resected oesophagus or regional lymph nodes was found, corresponding to a pathological complete response (pCR) rate of 25%. The pathological stages of the other resection specimens were: pT1N0-1M0 in 12 patients (23.1%), pT2N0-1M0 in 6 patients (11.5%), pT3N0-1M0 in 16 patients (30.8%), pT0-3N0-1M1A in 4 patients (7.7%), and pT1N1M1B in 1 patient (1.9%). In 19 patients (36.5%) a regression grade III, in 14 patients (26.9%) a regression grade II, and in 6 patients (11.5%) a histopathological regression grade I was seen. In 7 of the 18 patients (38.9%) with a pathological T3-stage only scattered tumour cells were found in the resection specimen. A radical resection with no evidence of tumour cells at the resection margins (R0-resection) was obtained in all patients. The lymph node dissection status showed a median of eight nodes (range 0–30), derived from both regional and distant sites. In 13 patients (25%) one or more positive lymph nodes were found (median 2, range 1–6). The N-stage improved from N1, as assessed by EUS, to N0 postoperatively in 19 patients (36.5%). In four patients (7.7%) the N0-stage, as assessed by EUS, was changed towards a N1-stage postoperatively.

### Pulmonary toxicity

The post-treatment pulmonary function tests (measured 6 months and 1 year after surgery) deteriorated significantly compared to the pretreatment tests. The total lung capacity (TLC) decreased from 103% of the predicted value to 92% (*P*=0.002). The vital capacity (VC) declined from 105% of the predicted value to 96% (*P*<0.001). The forced expiratory volume in 1 s (FEV1) decreased from 94% of the predicted value to 87% (*P*<0.0001). This decline in pulmonary function tests did not lead to major clinical symptoms.

### Survival

**Figure 1:**
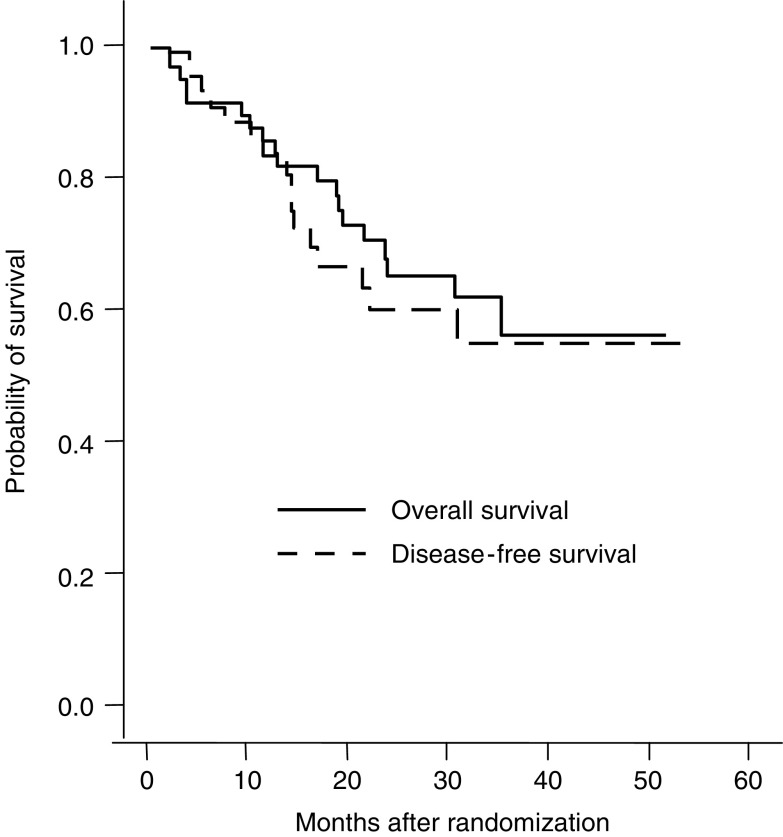
Kaplan–Meier survival curves.

All 54 patients were included in the survival analysis. At the time of evaluation (31 May 2005) the median follow-up time for all patients was 23.5 months (range 0–52 months). The median follow-up time for surviving patients was 31 months (range 11–52 months). Nineteen of the 54 patients (35.2%) died: 13 due to recurrent cancer, 5 during treatment (four postoperatively and one sudden death) and 1 due to a ruptured aortic aneurysm. The median survival time, however, has not yet been reached. The estimated 1-, 2-, and 3-year survival rates were 82, 65, and 56%, respectively. The Kaplan–Meier curve for overall survival is shown in Figure 1. The survival of patients with a pCR was not better than the survival of patients with no pCR. Recurrent disease after surgery was found in 15 patients surviving postoperatively (15/48, 31.2%). Three of them were still alive at the time of analysis. Recurrence was locoregional in seven patients. Distant metastases were found in 14 patients. The patient who refused surgery died from recurrent disease as well (see before). The Kaplan–Meier curve for disease-free survival of the patients surviving postoperatively is shown in Figure 1. The patient who died without recurrence was censored at the time of death.

## DISCUSSION

Preoperative chemoradiotherapy is nowadays widely used in the treatment of patients with potentially resectable oesophageal cancer. The concept that preoperative chemoradiotherapy may lead to a better tumour control and therefore to a better overall survival is appealing, as 29–43% incomplete resections are performed when patients are treated with surgery alone or with chemotherapy followed by surgery (Kelsen *et al*, 1998; Hulscher *et al*, 2002; MRC, 2002). Many studies have reported that after chemoradiotherapy in 10–28% of the patients no tumour cells are found in the resection specimen (Apinop *et al*, 1994; Le Prise *et al*, 1994; Walsh *et al*, 1996; Bosset *et al*, 1997; Urba *et al*, 2001). However, surprisingly few phase III studies have been reported in which preoperative chemoradiotherapy followed by surgery was compared with surgery alone. Meta-analyses of these trials showed a small, if any, effect on survival (Kaklamanos *et al*, 2003; Urschel and Vasan, 2003; Fiorica *et al*, 2004). In addition, the results of a recently reported study were disappointing, showing no survival benefit for those patients treated with preoperative chemoradiotherapy (Burmeister *et al*, 2005). In most studies, the combination of 5-FU and cisplatin with radiotherapy has been applied. In our study we used paclitaxel and carboplatin with concurrent radiotherapy.

Our study showed that preoperative chemoradiotherapy with weekly paclitaxel and carboplatin was well tolerated. All patients completed the chemoradiotherapy as scheduled, without treatment delay or dose reduction. The major non-haematological toxicity was a grade 3 or 4 oesophagitis in 7.5% of the patients. Compared to other studies with chemoradiotherapy in oesophageal cancer, this incidence of grade 3 and 4 oesophagitis is low (Urba *et al*, 2001; Choi *et al*, 2004).

The postoperative mortality of this study (7.7%; 95% CI 0–15%) was somewhat higher than the approximately 4% mortality rate found in other trials performed at our institution (van Lanschot *et al*, 2001; Hulscher *et al*, 2002; Polee *et al*, 2003); however, the observed mortality rate still lies within the 95% confidence limits. Postoperative morbidity consisted mainly of pulmonary complications. This high pulmonary complication rate is partly due to the fact that we also scored minor pulmonary complications, such as upper airway infections. Whether preoperative chemoradiotherapy is responsible for a higher pulmonary complication rate cannot be excluded. In a retrospective study of Avendano *et al* (2002) preoperative chemoradiotherapy was associated with an increase risk of pulmonary complications (i.e., duration of mechanical ventilation). Whether the decline in pulmonary function tests (TLC, VC, and FEV1) that we observed in our study was due to the chemoradiotherapy is uncertain, as it has also been reported that the TLC and the VC were significantly reduced after an oesophagectomy without preoperative treatment (Crozier *et al*, 1992).

During follow-up, 22 patients required dilatations because of an anastomotic stricture. The dilatations resolved the dysphagia in all patients, eventually all patients had an adequate food intake. Neoadjuvant chemoradiotherapy has not been reported to be associated with a higher stricture formation rate (Kelley *et al*, 2004).

In this study, the overall and disease-free survivals compare favourably with those in other trials of preoperative chemoradiation for oesophageal cancer. With a median follow-up of 23.5 months, the median survival time has not yet been reached. However, such findings should be interpreted with caution, because phase II studies always carry the risk of selection bias. A complete (R0) resection was accomplished in all patients, using a 1 mm cutoff point for circumferential resection margin and this also compares favourably with other studies. The pathologically complete response rate of 25% in our study is consistent with that in other studies using preoperative chemoradiation. A major histomorphological regression was seen in another 19 resected specimens. Thus, in a total of 32 patients (61.5%) a major or complete pathological response to preoperative chemoradiation was found. Several studies have shown that a pCR and an R0-resection are associated with a better prognosis (Hofstetter *et al*, 2002; Berger *et al*, 2005). Surprisingly, we were not able to demonstrate a significant survival difference between patients who had a pCR and those who did not have a pCR. As all operated patients had an R0 resection, a possible adverse effect of an incomplete resection on survival could not be assessed. The 100% complete resection rate and high number of patients with a major or complete pathological response might explain the lack of survival benefit in patients who had a pCR.

In conclusion, this study shows that preoperative treatment with weekly paclitaxel and carboplatin with concurrent radiotherapy is well tolerated, with leucopaenia and oesophagitis being the most common side effects. After chemoradiotherapy a high rate of radical resections could be achieved and the overall survival looks promising. A randomised phase III trial with this regimen followed by surgery *vs* surgery alone is now ongoing, which has, up to now, included more than 100 patients in the first year, to determine its role in the treatment of resectable oesophageal carcinoma.

## Figures and Tables

**Table 1:** Patient characteristics

**Characteristics**	**No. (%)**
Total no. of patients	54
	
*Sex*	
Male	49 (91)
Female	5 (9)
	
*Age (years)*	
Median	59
Range	40–75
	
*Performance status (ECOG)*	
0	35 (65)
1	18 (33)
Unknown	1 (2)
	
*Weight loss* (%)^a^	
Median	2
Range	0–12
	
*Histology*	
Adenocarcinoma	41 (76)
Squamous cell carcinoma	12 (22)
Large cell carcinoma	1 (2)
	
*Barrett's oesophagus* b	
Yes	19 (46)
No	17 (42)
Uncertain	5 (12)
	
*Stage (EUS)*	
T2N0	5 (9)
T2N1	2 (4)
T3N0	18 (33)
T3N1	21 (39)
No pass	8 (15)
	
*Primary site*	
Thoracic oesophagus	5 (9)
Lower oesophagus	49 (91)

a Calculated from the data of 52 patients.

b Calculated in 41 patients with adenocarcinoma. Yes=Barrett's oesophagus identified by upper endoscopy and confirmed by histopathologic examination. No=No Barrett's oesophagus identified by upper endoscopy or by histopathologic examination. Uncertain=Barrett's oesophagus identified by upper endoscopy or by histopathologic examination.

EUS, Endoscopic ultrasound.

**Table 2:** Haematological toxicities

	**Grade**				
	**0**	**1**	**2**	**3**	**4**
**(%)**	**No. (%)**	**No. (%)**	**No. (%)**	**No. (%)**	**No. (%)**
Anaemia	7 (13.2)	42 (79.2)	4 (7.5)	—	—
Leucopaenia	4 (7.5)	11 (20.8)	25 (47.2)	12 (22.6)	1 (1.9)
Neutropaenia	17 (32.1)	19 (35.8)	9 (17)	8 (15.1)	—
Thrombocytopaenia	30 (56.6)	19 (35.8)	3 (5.7)	1 (1.9)	—

Data from 53 evaluable patients.

**Table 3:** Non-haematological toxicities

	**Grade**				
	**0**	**1**	**2**	**3**	**4**
**(%)**	**No. (%)**	**No. (%)**	**No. (%)**	**No. (%)**	**No. (%)**
Nausea	15 (28.3)	35 (66)	3 (5.7)	—	—
Vomitus	36 (67.9)	15 (28.3)	2 (3.8)	—	—
Oesophagitis	11 (20.8)	23 (43.4)	15 (28.3)	4 (7.5)	—
Lethargy	23 (43.4)	23 (43.4)	7 (13.2)	—	—
Skin toxicity	34 (64.2)	18 (34)	1 (1.9)	—	—

Data from 53 evaluable patients.

**Table 4:** Postoperative complications

**Complication**	**No. of patients**
None	14
	
*Pulmonary*	22^a^
Upper airway infection	9
Pneumonia	7
Chylothorax	3
Pulmonary embolism	2
Pleural effusion	2
Atelectasis	1
Pleural empyema	1
Acute respiratory distress syndrome	1
	
*Cardiac*	12
Atrial fibrillation	10
Decompensatio cordis	2
Asystole during intubation	1
	
Wound infection	5
Vocal cord paralysis	3
Other	9

Data from 52 evaluable patients.

a In four patients two events.
